# The effect of physical activity intervention on blood pressure in 18 low and middle-income countries: a systematic review and meta-analysis of randomized controlled trials

**DOI:** 10.1186/s40885-024-00281-w

**Published:** 2024-08-01

**Authors:** Vahid Monfared, Mohtaram Hashemi, Fatemeh Kiani, Reyhane Javid, Mahsa Yousefi, Mahdis Hasani, Ali Jafari, Mohammad Ali Vakili, Motahareh Hasani

**Affiliations:** 1 Skeletal Biology Laboratory, College of Health, Oregon State University, Corvallis, OR 97331 USA; 2 Student Research Committee, Semnan University of Medical Sciences, Semnan, Iran; 3 Student Research Committee, Golestan University of Medical Sciences, Gorgan, Iran; 4 Department of Physical Education, Farhangian University, Tehran, Iran; 5 Student Research Committee, Department of Nutrition, School of Health, Golestan University of Medical Sciences, Gorgan, Iran; 6 Department of Biostatistics and Epidemiology, Health Management and Social Development Research Center, Faculty of Health, Golestan University of Medical Sciences, Gorgan, Iran; 7 Health Management and Social Development Research Center, Golestan University of Medical Sciences and Health Services, Hirkan Boulevard, Gorgan, 4918936316 Iran

**Keywords:** Blood pressure, Physical activity, Middle- and low-income nations

## Abstract

**Background:**

In especially, low and middle-income nations (LMICs), where healthcare access may be restricted, high blood pressure (BP) is a major risk factor for cardiovascular disease and stroke, both of which can even lead to death. Altering one's lifestyle, in conjunction with medical therapy, has been demonstrated to be effective in lowering BP. Recent research has shown that physical activity (PA), in a variety of guises and to varying degrees, can be an effective means of lowering BP.

**Objective:**

The purpose of this meta-analysis and systematic review was to evaluate the impact that PA plays in the development of hypertension in LMICs nations.

**Methods:**

An exhaustive search of the available research was carried out in order to locate studies that were pertinent. We searched a number of online databases, such as SCOPUS, Medline, and Web of Science, looking for clinical trials that were published before March of 2023. Studies were only considered for inclusion if they were randomized controlled trials (RCTs), reported on the association between PA and BP, and were carried out in LMICs countries.

**Results:**

This meta-analysis incorporated a comprehensive collection of 60 studies, encompassing a total of 11,002 people, consisting of 5,630 cases and 5372 controls. The findings indicate that engaging in PA had a notable impact on decreasing systolic blood pressure (SBP), as seen by a weighted mean difference (WMD) of -7.70 mmHg, with a 95% confidence interval (CI) ranging from -9.50 to -5.91 (*p* < 0.001). Additionally, PA was found to have a significant influence on reducing diastolic blood pressure (DBP), as indicated by a WMD of -3.60 mmHg, with a 95% CI ranging from -4.48to -2.73(*p* < 0.001). The findings from subgroup analysis indicate that the observed results remained statistically significant when considering individuals with baseline SBP of 120 mmHg or lower and DBP of 80 mmHg or lower.

**Conclusion:**

The incorporation of PA can significantly contribute to the mitigation of high BP within LMICs nations. Additional investigation is required to ascertain the most effective form and amount of PA in order to mitigate BP levels within these specific individuals.

**Graphical Abstract:**

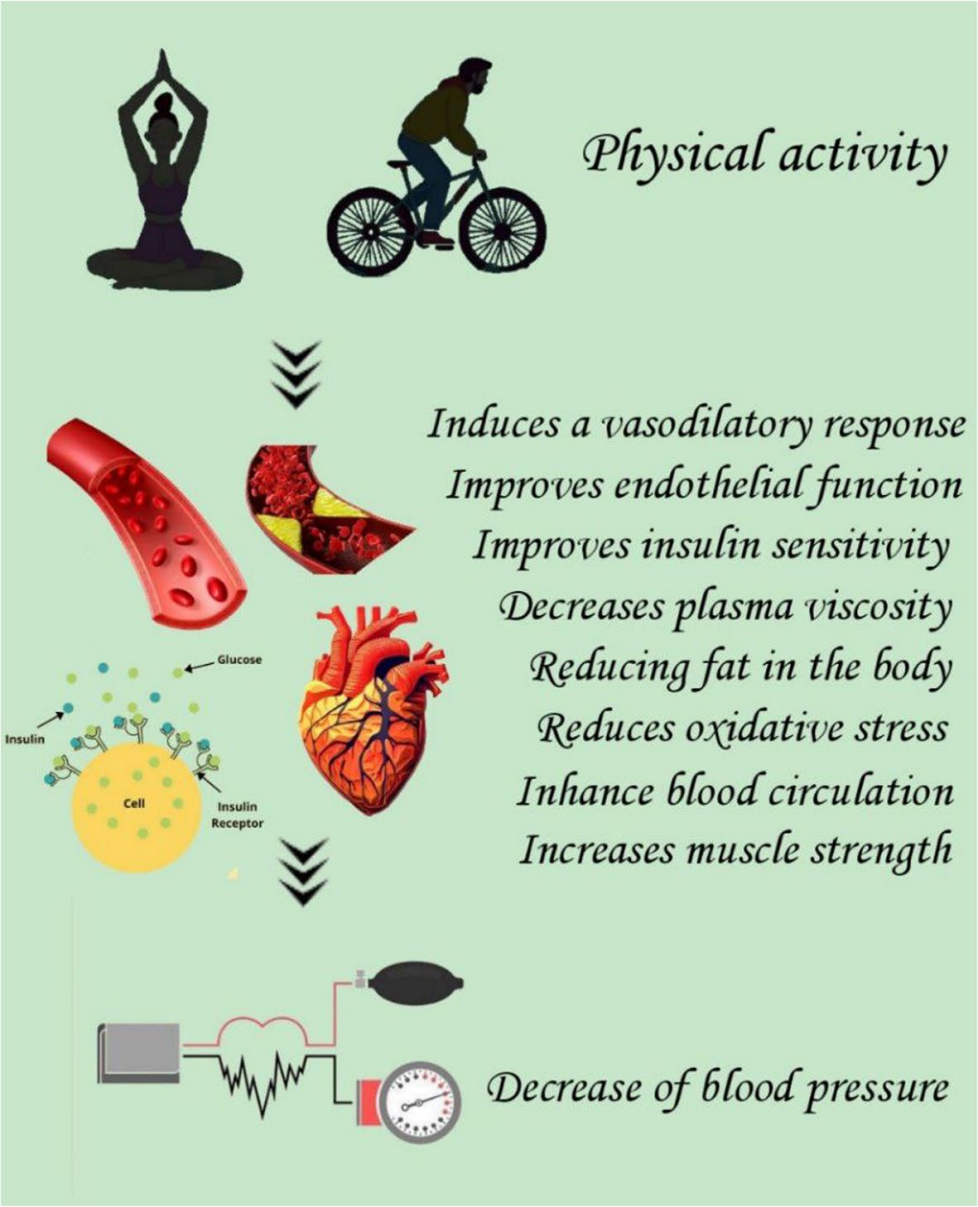

**Supplementary Information:**

The online version contains supplementary material available at 10.1186/s40885-024-00281-w.

## Introduction

Hypertension is diagnosed when an individual's systolic blood pressure (SBP) is equal to or greater than 140 mmHg, and/or their diastolic blood pressure (DBP) is equal to or greater than 90 mmHg [1]. It is a major contributor to global mortality, affecting 40% of adults and is linked to an increased risk of strokes and heart disease [2–7]. Up to 10 million deaths worldwide can be attributed to arterial hypertension [8]. In fact, a reduction in SBP, at least 10 mmHg, can decrease the risk of CVD by about 20–30 percent [2]. Various studies have examined symptoms of hypertension such as headaches, hot flushes, and mood disorders. Hypertension is often seen as asymptomatic, but these symptoms may indicate secondary hypertension caused by another medical condition [1, 9–11]. The prevalence of hypertension among adults is higher in low and middle-income countries (LMICs) in comparison to high-income nations [12]. Records from 2019 shows the highest normalized death rate caused by cardiovascular disease and SBP with regards to age in LMICs [13]. The World Health Organization (WHO) presented a table from The World Bank that categorizes countries as LMICs based on Gross National Income (GNI). A nation that possesses GNI per capita of $1,135 or lower is classified as a low-income country, whereas a nation with a GNI per capita ranging from $1,136 to $13,846 falls into the category of a middle-income country [14, 15]. According to the data presented in the table, there are a total of 26 countries classified as low-income and 108 countries classified as middle-income [14]. Patients with hypertension require a combination of lifestyle modifications and medication for effective medical care and self-management [16, 17]. WHO has just published a comprehensive inventory of indispensable pharmaceuticals for the management of hypertension. This list encompasses a range of medications, including angiotensin-converting enzyme (ACE) inhibitors, calcium channel blockers (CCBs), angiotensin receptor blockers (ARBs), and diuretics. In addition to the aforementioned considerations, it is imperative to address the management of other cardiovascular risk factors, such as smoking, diabetes, and lipid abnormalities [18–22]. Engaging in healthy lifestyle practices, including the maintenance of a normal BMI and waist circumference, regular physical activity, abstaining from smoking, moderate alcohol consumption, adherence to the DASH diet, and utilization of dietary supplements such as garlic, cocoa, vitamin C, coenzyme Q10, omega‐3 fatty acids, calcium, potassium, and magnesium, has been associated with favorable health outcomes among individuals diagnosed with hypertension [17, 23–28]. Moreover, lifestyle modifications may hold greater significance than pharmacological interventions for those with moderate hypertension. Thus, exercise training and other non-pharmacologic treatments should be recommended as the primary treatment for stage 1 hypertension in conjunction with medication [17]. Due to the significant health issues caused by hypertension and the lack of comprehensive studies. Therefore, a comprehensive systematic review and meta-analysis of clinical trial studies was undertaken to aggregate existing evidence pertaining to the correlation between physical activity intervention and blood pressure.

## Materials and methods

The PRISMA procedure, which is used for reporting systematic reviews and meta-analyses, served as the basis for the planning, execution, and reporting of this work [29].

### Information sources and search strategy

**Fig. 1:**
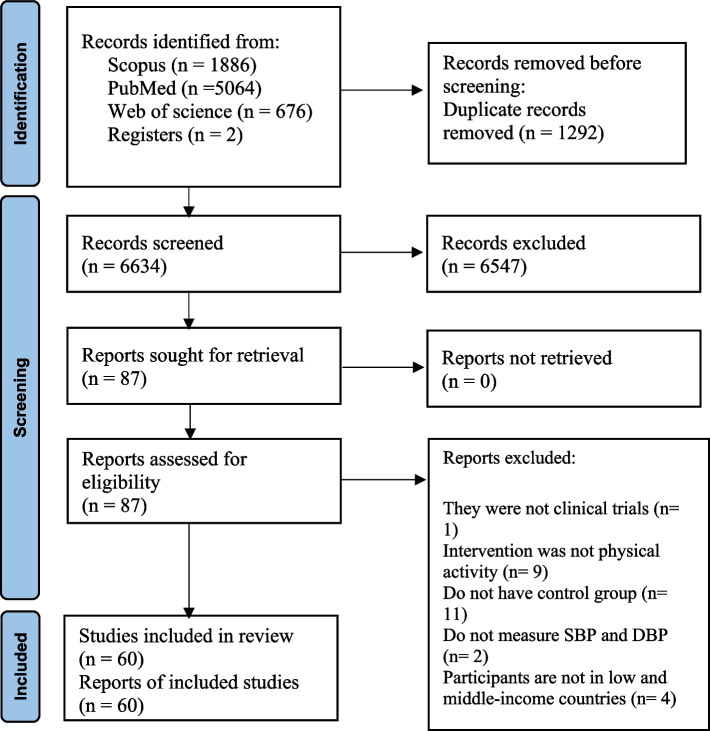
Flow diagram of the selection of the included studies

Online databases including SCOPUS (http://www.scopus.com), Medline (http://www.ncbi.nlm.nih.gov/PubMed), and Web of Science (https://clarivate.com › scientific-and-academic-research) were searched to find all the relevant clinical trials up to March 2023. We used the following search terms in our search in the mentioned databases: (Exercise OR "Exercise therapy" OR "Exercise test" OR "Exercise Movement Techniques" OR kinesiotherapy OR "Physical Endurance" OR Anaerobic OR aerobic OR "Resistance Training" OR "Motor activity" OR "Physical Activity" OR "Locomotor Activity" OR "relaxation therapy" OR tai-ji OR yoga) AND ("Blood pressure" OR Hypertension OR "Systolic Pressure" OR "Diastolic Pressure" OR "Pulse Pressure") AND ("Developing Country" OR "Under Developed country" OR "Underdeveloped country" OR "less Developed country" OR "Developing nation" OR "Under Developed nation" OR "Underdeveloped nation" OR "less Developed nation" OR "Third World" OR "low resource country" OR "low resource nation" OR Africa OR Africa OR "South America" OR "South America" OR "Latin America" OR "central America" OR Asia). All of the studies that were looked up were incorporated into the Endnote software (version X9, for Windows, Thomson Reuters, Philadelphia, PA, USA) and screened. After that, duplicate citations were removed from the articles. The present meta-analysis did not take into account information from unpublished sources or those considered as grey literature, such as conference abstracts, theses, and patents. In addition to this, we also conducted a hand search of the reference lists of the papers that were obtained as well as the reviews that were done previously in order to include any other studies that might be suitable. There were no limitations on language and date. The detailed steps of the literature search are depicted in (Fig. 1).

### Inclusion criteria

Human trials were included in the meta-analysis if they fulfilled the following inclusion criteria: (A) Were RCTs with either parallel or crossover designs; (B) Reported blood pressure indices before and after intervention in each group; (C) Compared intervention group with the placebo group; (D) Reported data as mean and SD or can be converted to it; (E) Were listed in LMICs according to the World Bank incoming group list used by WHO (Supplementary file).

### Exclusion criteria

Studies were excluded if they: (A) Were non clinical trials; (B) Duplicate studies (C) Animal, in vitro, and review studies; (D) Pregnant women; (E) Did not provide effects sizes on blood pressure before and after the trial in placebo and intervention groups; (F) They were conducted solely in developed (high-income) countries according to the World Bank incoming group list used by WHO.

### Data extraction

The following data were extracted with a standardized data collection form by three researchers working independently (Hashemi M, Javid R, Kiani F). After reviewing the titles and abstracts of the studies, relevant research was incorporated into the present study. The disagreements that arose amongst the reviewers were settled through discussion and by coming to an agreement with the assistance of a fourth reviewer (Monfared V). The following information was extracted: first author, publication year, mean age, gender, study design, sample size, intervention type, duration of intervention, mean and SD in BMI, health status, and mean and SD changes in SBP and DBP. The corrected mean changes and standard deviations of blood pressure measurements were calculated during the study for both the intervention group and the control group. If the data on the blood pressure were provided in a different unit, we converted them to the unit that was used the most frequently.

#### Risk of bias assessment

We applied the Cochrane quality assessment tool for assessing the risk of bias for each study included in the current meta-analysis [30]. This instrument comprised seven components, which included random sequence generation, allocation concealment, reporting bias, performance bias, detection bias, attrition bias, and additional sources of bias. A score of "high risk" was assigned to each domain if the study contained methodological concerns that could have affected its findings. A score of "low risk" was assigned if there was no imperfection for that domain, and a score of "unclear risk" was assigned if the information was insufficient to determine the impact of the study. If the trial met the criteria for "low risk" in all dimensions, then the study was regarded as being of excellent quality and carrying an extremely low risk of being biased overall. Both of the reviewers worked independently on the assessment of the potential for bias (Hashemi M and Yousefi M) (Table 2).

#### Statistical analysis

The overall effect sizes were calculated using the mean changes in blood pressure measurements and their respective standard deviations for both the intervention group and the control group. We were able to determine mean changes when they were not reported by considering the changes in blood pressure that occurred during the intervention. The standard deviation (SD) was determined in research projects that reported the standard error of means (SEM) by multiplying the SEM by the square root of the sample size, as follows: SD = SEM × √n. We used a random-effects model that considering differences from one study to the next in order to get an accurate picture of the overall effect sizes. The I^2^ statistic and the Cochrane's Q test were used to determine the presence of heterogeneity. It was determined that there was significant heterogeneity between studies if the I^2^ value was > 50% or *P* < 0.05 [31]. To find probable sources of heterogeneity, subgroup analyses were performed according to the predefined variables including Type of physical activities (Resistant training, Aerobic training, Combined exercise), Gender (Male, Female, Both), Baseline SBP (> 120 mmHg, ≤ 120 mmHg), Baseline DBP (> 80 mmHg, ≤ 80 mmHg), Age (> 50 years, ≤ 50 years), Health status (Healthy, Disease),Baseline BMI (Normal (18.5–24.9 kg/m^2^), Overweight (25–29.9 kg/m^2^), Obese (> 30 kg/m^2^)), Ethinc (Asia, Africa, America), and Trial duration (> 24 weeks, < 24 weeks) (Table 3). A sensitivity analysis was performed in order to determine whether or not the overall effect size was dependent on a specific study. The formal test developed by Begg looked into the possibility of publication bias. Stata, version 17/0 was utilized in the process of doing the meta-analysis, and *P* value < 0.05 was considered as significant level.

### Certainty assessment

The overall certainty of evidence across the studies was graded according to the GRADE guidelines Working Group [32]. According to the relevant assessment standards, the standard of the evidence can be divided into the following four categories: high, moderate, low, and extremely low. Monfared V and Hasani M, two pairs of authors, independently utilized the GRADE assessment and then consensus to reach a single result (Table 4).

## Results

### Study selection

Out of a total of 7,626 articles obtained from three databases (Scopus, PubMed, and Web of Science), the primary search yielded 1,886 items from Scopus, 5,064 articles from PubMed, and 676 articles from Web of Science. After excluding 1,292 duplicate studies, a total of 6,634 unique articles remained for further analysis. Following the examination of titles and abstracts, a total of 6,547 studies that did not meet our inclusion criteria were excluded. These exclusions were based on the following reasons: 5,779 studies had titles and abstracts that were irrelevant to our research topic, 465 studies had animal subjects, and 222 studies were review articles. As a result, a total of 87 studies were retained for analysis. Following a thorough examination of the full-text articles, one study was excluded due to its lack of a clinical trial design. Additionally, in nine studies, the intervention did not involve physical activity. Furthermore, 11 studies lacked an appropriate control group. Two study failed to measure SBP and DBP, while four studies did not include participants from LMICs countries. Ultimately, a total of 60 studies were identified that satisfied all the specified inclusion criteria and were then included in the meta-analysis. Figure 1 illustrates the PRISMA flow diagram, which outlines the search process.

### Study characteristics

Overall, 60 studies, with 11,002participants (5,630cases and 5,372controls), were included [33–94]. Included studies were published between 2002 [58] and 2021 [66]. The duration period ranged from 2 [55] days to 96 [54] weeks, and the sample size of the included studies ranged from 20 to [56] to 674 [54] participants. The mean age ranged from 13 [67] to 71 [40], and BMI ranged from 20 [67] to 32 [34] All of the studies were parallel RCTs, except for three studies that were cross-over designs [52, 53, 55]. Selected studies enrolled subjects with hypertension [36, 40, 47, 48, 50, 52–56, 58, 59, 62, 71–75, 86–89],metabolic syndrome [44, 51, 69, 80, 82], healthy persons [34, 35, 37, 49, 60, 61, 63, 65–67, 70, 76, 78, 83, 84, 91, 94], type 2 diabetes mellitus [46, 64, 90, 92], peripheral artery disease [68], symptomatic peripheral artery disease [81], chronic hemodialysis patient [33], Parkinson disease [39], Prehypertension [42], HIV/AIDS [85], chronic heart failure [41], coronary artery disease [43], Stable coronary artery disease [79], coronary heart disease [38], cardiovascular risk [45, 93]. The investigations were conducted in various countries, including the India [43, 59, 78, 83, 90], China [35, 37, 38, 44, 47, 51, 60, 63, 65, 66, 79], Iran [36, 70, 76], Sri Lanka [46], Brazil [39, 50, 55, 56, 67, 68, 81, 86–88, 91, 92, 94, 95], Taiwan [40, 48, 61, 62, 82, 93, 95], East Africa [34, 49], South Africa [64], Tunisia [33], Colombia [45, 69], Thailand [71], Nigeria [41, 52, 53, 72–75, 85], one multinational study in (Argentina،Guatemala، Peru) [42], Egypt [84], Chile [80], and Pakistan [54]. Characteristics of the included studies are abstracted in Table 1.

**Table 1:** Characteristic of included studies in meta-analysis

Studies	Year	Country	Study Design	Participant		Sample size and Sex	Sample size		Trial Duration (Week)	Means Age		Means BMI		Health Status
				**IG**	**CG**		**IG**	**CG**		**IG**	**CG**	**IG**	**CG**	
**Mandal et al**	2021	India	RCT	Yoga program (asana, pranayama, and deep relaxation technique)	No intervention	110, Both	58	52	12	35 ± 7.9	32.5 ± 6.8	25.9 ± 4.2	24.4 ± 4.4	Healthy
**Yu et al**	2021	China	CT	Brisk walking level 1	Daily walking	277, Both	92	185	12	37.0 ± 9.9	37.0 ± 9.9	23.9 ± 3.9	23.9 ± 3.9	Healthy and sedentary lifestyle
**Yu et al**	2021	China	CT	Brisk walking level 2	Daily walking	359, Both	174	185	12	37.0 ± 9.9	37.0 ± 9.9	23.9 ± 3.9	23.9 ± 3.9	Healthy and sedentary lifestyle
**Yu et al**	2021	China	CT	Brisk walking level 3	Daily walking	422, Both	237	185	12	37.0 ± 9.9	37.0 ± 9.9	23.9 ± 3.9	23.9 ± 3.9	Healthy and sedentary lifestyle
**Gu et al**	2020	China	Prospective self- CT	Walking and self-monitoring by a pedometer	No Intervention	262, Both	150	112	100 DAY	NR	NR	23.7 ± 3.0	22.7 ± 3.1	Healthy
**Hooshmand-moghadam et al**	2020	Iran	RCT	Resistance training (RT)	No Intervention	24, Male	12	12	18	63.2 ± 8.09	62.5 ± 10.25	28.2 ± 2.25	28.4 ± 2.25	Hypertension
**Hooshmand-moghadam et al**	2020	Iran	RCT	RT and Saffron (RTS)	No Intervention	24, Male	12	12	18	63.5 ± 8.09	62.5 ± 10.25	28.4 ± 2.25	28.4 ± 2.25	Hypertension
**Ranasinghe et al**	2020	Sri Lanka	RCT	Aerobic Exercise Training	Standard Care	58, Both	28	30	12	52 ± 9.8	49.3 ± 7.0	26.77 ± 4.23	25.85 ± 4.38	Type 2 Diabetes Mellitus
**Ranasinghe et al**	2020	Sri Lanka	RCT	Resistance Exercise Training	Standard Care	58, Both	28	30	12	49.0 ± 9.2	49.3 ± 7.0	26.86 ± 4.23	25.85 ± 4.39	Type 2 Diabetes Mellitus
**Rosa Santos et al**	2020	Brazil	RCT	Resistance training	No intervention	122, Both	61	61	12	13.39 ± 0.97	13 ± 1.04	20.30 ± 2.99	18.39 ± 2.76	Healthy
**Ac et al**	2019	Brazil	RCT	Isometric handgrip training (IHT)	Placebo	79, Both	29	50	8	66 ± 12	67 ± 11	27 ± 5	26 ± 7	Peripheral artery disease
**Ac et al**	2019	Brazil	RCT	Isometric handgrip training (IHT)	Placebo	73, Both	27	46	8	66 ± 12	67 ± 11	27 ± 5	26 ± 7	Peripheral artery disease
**Ac et al**	2019	Brazil	RCT	Isometric handgrip training (IHT)	Placebo	102, Both	50	52	8	66 ± 12	67 ± 11	27 ± 5	26 ± 7	Peripheral artery disease
**Ac et al**	2019	Brazil	RCT	Isometric handgrip training (IHT)	Placebo	98, Both	47	51	8	66 ± 12	67 ± 11	27 ± 5	26 ± 7	Peripheral artery disease
**Leung et al**	2019	China	RCT	Tai Chi Exercise Program	Usual Daily Activities	35, Both	18	17	12	62.19 ± 5.93	65.52 ± 9.34	27.40 ± 4.82	27.25 ± 4.34	Metabolic Syndrome
**Mouodi et al**	2019	Iran	Population-based RCT	Nutrition and physical activity training classes (aerobic), individual nutrition consultation, educational package and weekly aerobic exercises	Educational package	203, Both	106	97	16	49.8 ± 5.2	49.5 ± 5.7	28.3 ± 4.3	28.2 ± 4.3	Healthy
**Mouodi et al**	2019	Iran	Population-based RCT	Nutrition training classes, individual nutrition consultation and educational package	Educational package	195, Both	98	97	16	49.8 ± 5.4	49.5 ± 5.7	28.2 ± 4.6	28.2 ± 4.3	Healthy
**Chhugani et al**	2018	India	A Controlled Pilot Study	Integrated Yoga (IY)	No Intervention	30, Female	17	13	4	34 ± 8.4	34 ± 8.4	NR	NR	Healthy
**Gradidg et al**	2018	South Africa	RCT	Physical activity (walking)	No Intervention	115, Female	49	66	12	NR	NR	32.1 ± 6.61	31 ± 6.57	Healthy
**Ma et al**	2018	China	CT	Tai Chi Training	Usual Care	158, Both	79	79	24	70.24 ± 10.25	69.71 ± 10.84	26.01 ± 3.38	25.96 ± 2.91	Hypertension
**Cavalcante et al**	2017	Brazil	Prospective, controlled, crossover	Arm crank Exercise	Control session	11, Male	4	7	NR	64 ± 8	64 ± 8	25.05	25.05	Symptomatic peripheral artery disease
**Frih et al**	2017	Tunisia	RCT, SB	Resistance and endurance training exercises	No Intervention	41, Male	21	20	16	64.2 ± 3.4	65.2 ± 3.1	25.4 ± 2.8	24.3 ± 3.2	Chronic hemodialysis patient
**Kanegusuku et al**	2017	Brazil	RCT	Resistance Training	Usual Lifestyle	27, Both	15	12	12	67 ± 8	63 ± 8	26.3 ± 3.5	25.9 ± 4.3	Parkinson disease
**Kanegusuku et al**	2017	Brazil	RCT	Resistance Training	Usual Lifestyle	31, Both	15	16	12	67 ± 8	68 ± 10	26.3 ± 3.5	24.8 ± 3.0	Parkinson disease
**Chang et al**	2016	Taiwan	RCT	Community-based program (provided exercise environments, exercise skills and volunteers’ reminding)	No Intervention	131, Both	65	66	24	55.41 ± 14.05	55.08 ± 14.40	29.95 ± 3.11	30.42 ± 2.59	Obese individuals (BMI ≥ 27 kg/m2) with Metabolic syndrome
**Farinatti et al**	2016	Brazil	CT	Home-based exercise programs (light- to moderate-intensity walking and complementary stretching exercises three times per week)	No Intervention	43, Both	29	14	64	53 ± 11	48 ± 5	30.5 ± 4.6	30.4 ± 4.5	Hypertension
**Gallo-Villegas et al**	2016	Colombia	RCT	Aerobic exercise at 3 times a week, muscle strength training twice a week, and the nutrition education program 2-h workshops each week	General recommendations about exercise and healthy eating	59, Both	30	29	12	49.2 ± 8.7	52. ± 6.8	29 ± 3	28.3 ± 4	Metabolic syndrome
**Rubinstein et al**	2016	Argentina،Guatemala، Peru	RCT	Mobile phone-based health intervention (physical activity and diet)	Usual Care	553, Both	266	287	48	43.6 ± 8.4	43.2 ± 8.4	30.2 ± 5.2	30.8 ± 5.3	Prehypertension
**Lau et al**	2015	China	RCT	Hatha Yoga Intervention	Routine Activities and not to Begin any Exercise	87, Both	44	43	12	52.44 ± 7.15	51.52 ± 7.78	24.44 ± 3.84	25.90 ± 3.90	with Metabolic Syndrome
**Lau et al**	2015	China	RCT	Hatha Yoga Intervention	Routine Activities and not to Begin any Exercise	86, Both	43	43	12	52.44 ± 7.15	51.52 ± 7.78	24.44 ± 3.84	25.90 ± 3.90	without Metabolic Syndrome
**Lau et al**	2015	China	RCT	Hatha Yoga Intervention	Routine Activities and not to Begin any Exercise	173, Both	87	86	12	52.44 ± 7.15	51.52 ± 7.78	24.44 ± 3.84	25.90 ± 3.90	with/without Metabolic Syndrome
**Ezema et al**	2014	Nigeria	RCT	Moderate intensity continuous exercise training + Conventional therapy	Conventional therapy	30, Both	15	15	8	40.07 ± 9.72	32.47 ± 10.41	28.65 ± 7.11	29.01 ± 4.42	HIV/AIDS
**Sikiru et al**	2014	Nigeria	Cross over, DB, RCT	Bicycle ergometer at a low intensity, 8 weeks of continuous training, 3 times per week	Remain Sedentary	217, Male	112	105	8	58.63 ± 7.22	58.27 ± 6.24	22.48 ± 2.89	24.16 ± 4.91	Hypertension
**Sujatha et al**	2014	India	RCT	Yoga program	No intervention	238, Both	118	120	12	NR	NR	27.61 ± 4.96	27.29 ± 5.28	Hypertension
**Wu et al**	2014	China	RCT	Lifestyle: diet and moderate-intensity aerobic or endurance-type activities, such as pulse-controlled brisk walking, jogging, dancing	Usual eating patterns and activities	100, Female	53	47	48	50.62 ± 3.92	49.06 ± 4.30	24.14 ± 2.70	24.33 ± 3.24	Healthy
**Yan et al**	2014	East Africa	RCT	low intensity exercise (LEX), vigorous-intensity exercise (VEX)	No intervention	41, Male	31	10	48	53 ± 2	55 ± 3	27.2 ± 0.7	27 ± 1.3	Type 2 diabetes mellitus
**Yu et al**	2014	China	Community-based CT	Lifestyle: improving physical activity (activity between 3 and 6 METs and lasting at least 6 min) and dietary patterns	Usual standard health care	273, Both	175	98	24	60.9 ± 10.2	60.1 ± 10	NR	NR	Older adults with diabetes and hypertension
**Hou et al**	2014	China	RCT	Chinese traditional healthcare exercises (CTHE)	No Intervention	136, Both	68	68	7	13.9	13.7	NR	NR	Healthy
**Ajiboye et al**	2013	Nigeria	RCT	Exercise Training (12 weeks of aerobic and resistance training 3 session per week)	Usual Drug Therapy	32, Both	17	15	12	54.1 ± 9.07	53.7 ± 11.6	30.4 ± 5.36	27 ± 7.35	Chronic Heart Failure
**Brito et al**	2013	Brazil	RCT	High-intensity resistance exercise (50%)	Control session	20, Female	10	10	NR	65.5 ± 3	65.5 ± 3	28.7 ± 3	28.7 ± 3	Hypertensive elderly
**Brito et al**	2013	Brazil	RCT	High-intensity resistance exercise (80%)	Control session	20, Female	10	10	NR	65.5 ± 3	65.5 ± 3	28.7 ± 3	28.7 ± 3	Hypertensive elderly
**Lamina et al**	2013	Nigeria	RCT	Aerobic exercise training program	Sedentary	245, Male	140	105	8	58.40 ± 6.91	58.27 ± 6.24	24.96 ± 3.88	24.16 ± 4.91	Essential hypertension
**Pal et al**	2013	India	RCT	Medication + Yoga	Medication Only	208, Both	105	103	72	59.1 ± 9.9	56.4 ± 10.9	24.8 ± 3.8	25.1 ± 4.8	Coronary artery disease
**Sikiru et al**	2013	Nigeria	Cross over, DB, RCT	Bicycle ergometer at a low intensity, 8 weeks of continuous training, 3 times per week	Remain Sedentary	245, Male	140	105	8	58.40 ± 6.91	58.27 ± 6.24	24.96 ± 3.88	24.16 ± 4.91	Mild to Moderate Hypertension
**Tsai et al**	2013	Taiwan	Non-randomized quasi-experimental design	Low intensity exercise	No intervention	59, Both	30	29	12	34.8 ± 7.0	33.3 ± 9.4	NR	NR	Healthy
**Tsai et al**	2013	Taiwan	Non-randomized quasi-experimental design	High intensity exercise	No intervention	59, Both	30	29	12	41.0 ± 7.2	33.3 ± 9.4	NR	NR	Healthy
**Lamina et al**	2012	Nigeria	RCT	Aerobic exercise training (bicycle ergometer)	No intervention	217, Male	112	105	8	58.63 ± 7.22	58.27 ± 6.24	22.92 ± 2.2	23.37 ± 3.87	Essential hypertension
**Lo et al**	2012	Taiwan	RCT	Yang-Style Tai Chi Exercise Program	Routine Care	58, Both	27	31	8	58.47 ± 7.46	58.47 ± 7.46	NR	NR	Hypertension
**M. Cunha et al**	2012	Brazil	Cross over،RCT	Aerobic and Water Exercise	Did not enter the pool and did not exercise	32, Female	16	16	2 DAYS	66 ± 2.94	66 ± 2.94	27.32 ± 4.30	27.32 ± 4.30	Hypertensive elderly women
**Mizuno et al**	2012	Brazil	RCT	Yoga Exercises	Gave Up Yoga Classes	33, Both	17	16	16	67 ± 7	62 ± 12	27.4 ± 4.4	26.4 ± 5.3	Arterial Hypertension
**Vianna et al**	2012	Brazil	CT	Aerobics exercises (walking, hydrogymnastics, weight-training exercises, and stretching exercises)	Was advised to keep their daily routines	70, Both	35	35	16	68.66 ± 5.93	69.8 ± 8.05	26.74 ± 3.81	26.09 ± 4.58	Healthy
**Kanegusuku et al**	2011	Brazil	RCT	Strength training	Normotensive older adults who did not take part in any training program	24, Both	13	11	16	63 ± 3.60	63 ± 3.31	27.4 ± 5.76	27.3 ± 4.97	Healthy
**Kanegusuku et al**	2011	Brazil	RCT	Strength training	Normotensive older adults who did not take part in any training program	24, Both	13	11	16	63 ± 3.60	63 ± 3.31	27.4 ± 5.76	27.3 ± 4.97	Healthy
**Kanegusuku et al**	2011	Brazil	RCT	Power training	Normotensive older adults who did not take part in any training program	26, Both	15	11	16	65 ± 3.87	63 ± 3.31	26.5 ± 4.64	27.3 ± 4.97	Healthy
**Kanegusuku et al**	2011	Brazil	RCT	Power training	Normotensive older adults who did not take part in any training program	26, Both	15	11	16	65 ± 3.87	63 ± 3.31	26.5 ± 4.64	27.3 ± 4.97	Healthy
**Lamina et al**	2011	Nigeria	RCT, DB	Exercise training (interval)	Sedentary	245, Male	140	105	8	58.40 ± 6.91	58.27 ± 6.24	24.96 ± 3.88	24.16 ± 4.91	Essential mild to moderate hypertension
**Lamina et al**	2011	Nigeria	RCT, DB	Exercise on a bicycle ergometer	No intervention	245, Male	140	105	8	58.63 ± 7.22	58.27 ± 6.24	22.48 ± 2.89	24.16 ± 4.91	Essential hypertension
**Lamina et al**	2011	Nigeria	RCT, DB	Exercise on a bicycle ergometer	No intervention	217, Male	112	105	8	58.40 ± 6.91	58.27 ± 6.24	24.96 ± 3.88	24.16 ± 4.91	Essential hypertension
**Luk et al**	2011	China	RCT	12 Each session would last for an hour with combined endurance and resistance exercise, beginning with warm-up stretching exercise	Non-exercise	64, Both	32	32	8	67.7 ± 9.0	66.6 ± 7.9	24.7 ± 2.4	25.1 ± 2.6	Stable coronary artery disease
**Mortimer et al**	2011	South Africa	RCT	Handgrip Training	Without Exercising	18, Female	9	9	5 DAYS	47.88 ± 5.4	49.88 ± 4.2	24.92 ± 3.9	27.26 ± 2.4	Healthy
**Ansari et al**	2010	Egypt	Quantitative study	One hour of moderate exercise three times a week for three months	No Intervention	90, Female	45	45	12	15.7 ± 1.8	15.4 ± 1.6	21.6 ± 4.5	21.4 ± 3.8	Healthy
**Ansari et al**	2010	Egypt	Quantitative study	One hour of moderate exercise three times a week for three months	No Intervention	70, Male	35	35	12	15.7 ± 1.8	15.4 ± 1.6	20.9 ± 4.1	21.2 ± 3.6	Healthy
**Monteiro et al**	2010	Brazil	CT	Aerobic training program	Attended educational lectures once a week	22, Female	11	11	13	61 ± 1.18	60.2 ± 0.4	27.5 ± 4	28.1 ± 6.61	Elderly women with type-2 diabetes mellitus
**Mujica et al**	2010	Chile	RCT	Exercise program (walking)	No intervention	51, Both	27	24	18	49.4 ± 6.2	51.1 ± 5.3	31.8 ± 4.0	29.6 ± 3.8	Metabolic syndrome
**Arora et al**	2009	India	RCT	Aerobic exercise	Went under no training	20, Both	10	10	8	52.2 ± 9.3	58.4 ± 1.8	26.23 ± 3.2	24.98 ± 3	With type 2 diabetes
**Arora et al**	2009	India	RCT	Progressive resistance training (PRT)	Went under no training	19, Both	9	10	8	49.6 ± 5.2	58.4 ± 1.8	26.99 ± 4.1	24.98 ± 3	With type 2 diabetes
**Bündchen et al**	2009	Brazil	RCT	Aerobic and resistance exercises 30 to 60 min 3 times a week	Did not participate in the exercise programs	111, Both	57	54	12	58 ± 8.9	60 ± 7.7	30.1 ± 5	32.3 ± 7	Hypertensive patients with overweight or obesity
**Jafar et al**	2009	Pakistan	Cluster RCT	General Practitioner (GP) AND Home Health Education (HHE)	No Intervention	658, Both	332	326	96	54 ± 11.5	53.3 ± 11.5	NR	NR	Hypertension
**Jafar et al**	2009	Pakistan	Cluster RCT	General Practitioner (GP) Education	No Intervention	661, Both	335	326	96	55.3 ± 11.5	53.3 ± 11.5	NR	NR	Hypertension
**Jafar et al**	2009	Pakistan	Cluster RCT	Home Health Education (HHE)	No Intervention	674, Both	348	326	96	52.7 ± 11.4	53.3 ± 11.5	NR	NR	Hypertension
**Barroso et al**	2008	Brazil	RCT	Non-pharmacological treatment and physical activity consisting of 1-h sessions, 3 times a week	Non-pharmacological treatment (NPT)	35, Both	22	13	24	66.5 ± 4	70.8 ± 6.3	NR	NR	With stage I hypertension who were not using antihypertensive medication
**Meirelles et al**	2008	Brazil	CT	Aerobic, three times a week for 12 weeks	Sedentary hypertensive patients on stage 1 not doing the exercise	19, Both	13	6	12	49 ± 1	50 ± 4	30 ± 1	32 ± 2	Sedentary hypertensive patients on stage 1
**Meirelles et al**	2008	Brazil	CT	Aerobic, three times a week for 12 weeks	Sedentary hypertensive patients on stage 1 not doing the exercise	19, Both	13	6	12	49 ± 1	50 ± 4	30 ± 1	32 ± 2	Sedentary hypertensive patients on stage 1
**Jiang et al**	2007	China	RCT	Walking Performance, Step II diet	Routine care	167, Both	83	84	24	62.11 ± 7.44	61.37 ± 7.61	NR	NR	Coronary Heart Disease Patients
**Lee et al**	2007	Taiwan	RCT	Walking intervention	Usual Primary Health Care	184, Both	91	93	24	71.3 ± 6.4	71.3 ± 5.7	25.4 ± 3.8	25.31 ± 3.5	Mild to Moderate Hypertension
**Pazoki et al**	2007	Iran	RCT	10 min per day of moderate-intensity physical activity and encouraged to do 30 min of physical activity daily and healthy eating	No intervention	335, Female	170	165	8	NR	NR	28.02 ± 4.74	27.82 ± 5.39	Healthy
**Wu et al**	2007	Taiwan	CT	Treadmill training program for 30 min each time, 3 times a week	Maintained their previous lifestyles	36, Female	18	18	8	49.7 ± 6.1	51.8 ± 6.4	28.4 ± 3.1	27.3 ± 3.7	Cardiovascular risk
**Mendivil et al**	2006	Colombia	RCT	Nutritional Intervention Program plus physical activity (Aerobic dancing, soccer, basketball, recreational kickboxing, and a few resistance activities to strengthen localized muscular groups)	Nutritional Intervention Program	49, Both	28	21	16	50.03 ± 8.09	53.04 ± 8.18	26.3 ± 4.72	27.5 ± 4.09	Cardiovascular risk
**McCaffrey et al**	2005	Thailand	CT	Yoga program	Typical outpatient teaching about hypertension	54, Both	27	27	8	56.7	56.2	25.74 ± 2.87	25.32 ± 3.19	Hypertensive persons
**Thomas et al**	2005	China	Longitudinal, randomized, controlled intervention	Tai Chi	Usual level of physical activity	142, Both	64	78	48	68.9 ± 2.8	69 ± 3	23.8 ± 3.9	24.2 ± 3	Healthy elderly subjects
**Thomas et al**	2005	China	Longitudinal, randomized, controlled intervention	Resistance training	Usual level of physical activity	143, Both	65	78	48	69.1 ± 3.2	69 ± 3	24.2 ± 3.8	24.2 ± 3	Healthy elderly subjects
**Tsai et al**	2003	Taiwan	RCT	Tai Chi Chuan exercise training program	Sedentary life	76, Both	37	39	12	51.6 ± 16.3	50.5 ± 9.8	23.8 ± 2.4	24.1 ± 1.8	Stage I hypertension
**Tsai et al**	2003	Taiwan	RCT	Tai Chi Chuan exercise training program 3 times per week	Maintained their usual lifestyle behaviors	76, Both	37	39	12	51.6 ± 16.3	50.5 ± 9.8	23.8 ± 2.4	24.1 ± 1.8	Stage I hypertension
**Tsai et al**	2002	Taiwan	RCT	Moderate-intensity exercise	No exercise	23, Both	12	11	12	49.6 ± 9.3	46.2 ± 5.6	26.1 ± 4.5	25 ± 1.8	Mild hypertensive patients

*Abbreviations*: *IG* Intervention group, *CG* Control group, *DB* Double-blinded, *CT* Control trial, *SB* Single-blinded, *PC* Placebo-controlled, *CO* Controlled, *RA* Randomized, *NR* Not reported, *F* Female, *M* Male, *NR* Not reported

### Quality assessment

The Cochrane scoring system was used to assess the quality of the included studies (Table 2). This system consists of seven criteria to evaluate the risk of bias, which are as follows: random sequence generation, allocation concealment, blinding of participants and personnel, blinding of outcome assessment, incomplete outcome data, selective reporting, and other biases. Bias was assessed (high, low, or unclear) for individual elements, interpreted as high risk, low risk, and unknown risk, respectively. We classified 34 studies as low risk [35–37, 41–43, 45–47, 49, 52–54, 56, 62, 63, 72–76, 79, 81–88, 90–95], 16 moderate risk [33, 34, 50, 58–60, 64–67, 69–72, 78, 80], and 10 high-risk studies [38–40, 44, 48, 51, 55, 61, 68, 79].

**Table 2:** Risk of bias assessment

Study	Random sequence generation	Allocation concealment	Selective reporting	Other sources of bias	Blinding (participants and personnel)	Blinding (outcome assessment)	Incomplete outcome data	General risk of bias
Mandal et al., 2021 [78]	L	L	L	H	U	H	L	Moderate risk
Yu et al., 2021 [66]	U	L	L	H	U	H	L	Moderate risk
Gu et al., 2020 [35]	U	L	L	L	U	U	L	Low risk
Hooshmand-moghadam et al., 2020 [36]	L	L	L	L	U	U	L	Low risk
Ranasinghe et al., 2020 [46]	U	U	L	H	L	L	L	Low risk
Rosa Santos et al., 2020 [67]	U	L	L	H	U	H	L	Moderate risk
Ac et al., 2019 [68]	L	L	L	H	H	H	L	High risk
Leung et al., 2019 [51]	L	U	L	H	H	H	L	High risk
Mouodi et al., 2019 [76]	L	L	L	L	U	H	L	Low risk
Chhugani et al., 2018 [83]	U	U	L	H	U	U	L	Low risk
Gradidg et al., 2018 [34]	L	L	H	H	L	L	L	Moderate risk
Ma et al., 2018 [47]	U	U	L	H	U	U	L	Low risk
Cavalcante et al., 2017 [81]	L	H	L	L	U	L	L	Low risk
Frih et al., 2017 [33]	L	L	L	L	H	H	L	Moderate risk
Kanegusuku et al., 2017 [39]	U	H	H	H	H	H	H	High risk
Chang et al., 2016 [82]	L	L	L	H	U	U	L	Low risk
Farinatti et al., 2016 [86]	L	L	L	H	U	U	L	Low risk
Gallo-Villegas et al., 2016 [69]	L	L	L	L	H	H	L	Moderate risk
Rubinstein et al., 2016 [42]	L	L	L	L	H	U	L	Low risk
Lau et al., 2015 [44]	H	U	L	H	H	H	L	High risk
Ezema et al., 2014 [85]	L	U	L	H	U	U	L	Low risk
Sikiru et al., 2014 [53]	L	L	L	H	L	L	L	Low risk
Sujatha et al., 2014 [59]	L	L	L	H	U	H	L	Moderate risk
Wu et al., 2014 [63]	L	L	L	L	L	H	L	Low risk
Yan et al., 2014 [64]	U	L	L	H	U	H	L	Moderate risk
Yu et al., 2014 [65]	H	L	L	L	U	H	L	Moderate risk
Hou et al., 2014 [37]	L	U	L	L	U	U	L	Low risk
Ajiboye et al., 2013 [41]	L	U	L	H	U	U	L	Low risk
Brito et al., 2013 [56]	L	U	L	H	U	U	L	Low risk
Lamina et al., 2013 [75]	U	L	L	H	L	U	L	Low risk
Pal et al., 2013 [43]	L	L	L	H	U	U	L	Low risk
Sikiru et al., 2013 [52]	U	L	L	H	L	L	L	Low risk
Tsai et al., 2013 [61]	H	U	L	H	U	H	L	High risk
Lamina et al., 2012 [72]	L	L	L	H	U	H	L	Moderate risk
Lo et al., 2012 [48]	H	H	L	H	U	U	L	High risk
M. Cunha et al., 2012 [55]	U	H	L	H	H	H	L	High risk
Mizuno et al., 2012 [50]	H	U	L	H	U	U	L	Moderate risk
Vianna et al., 2012 [94]	L	U	L	L	U	U	L	Low risk
Kanegusuku et al., 2011 [91]	L	U	L	L	U	L	L	Low risk
Lamina et al., 2011 [73]	U	L	L	H	L	U	L	Low risk
Lamina et al., 2011 [74]	U	L	L	H	L	U	L	Low risk
Luk et al., 2011[79]	L	L	L	H	H	H	L	High risk
Mortimer et al., 2011 [49]	U	U	L	H	U	U	L	Low risk
Ansari et al., 2010 [84]	L	L	L	H	U	U	L	Low risk
Monteiro et al., 2010 [92]	L	U	L	H	U	U	L	Low risk
Mujica et al., 2010 [80]	L	U	L	H	L	H	L	Moderate risk
Arora et al., 2009 [90]	L	U	L	L	U	U	L	Low risk
Bündchen et al., 2009 [88]	L	U	L	H	U	U	L	Low risk
Jafar et al., 2009 [54]	L	L	L	L	L	L	L	Low risk
Barroso et al., 2008 [87]	L	U	H	L	U	U	L	Low risk
Meirelles et al., 2008 [89]	U	U	L	H	U	U	L	Low risk
Jiang et al., 2007 [38]	L	L	L	H	H	H	L	High risk
Lee et al., 2007 [40]	L	L	L	H	H	H	L	High risk
Pazoki et al., 2007 [70]	U	U	L	H	U	H	L	Moderate risk
Wu et al., 2007 [93]	U	U	L	H	U	U	L	Low risk
Mendivil et al., 2006 [45]	L	U	L	L	U	U	L	Low risk
McCaffrey et al., 2005 [71]	U	U	L	H	U	H	L	Moderate risk
Thomas et al., 2005 [60]	L	L	L	H	U	H	L	Moderate risk
Tsai et al., 2003 [62]	L	L	L	L	L	H	L	Low risk
Tsai et al., 2002 [58]	U	L	L	H	U	H	L	Moderate risk

General Low risk < 2 high risk

General moderate risk = 2 high risk

General high risk > 2 high risk

### Effect of physical activity on SBP

**Fig. 2:**
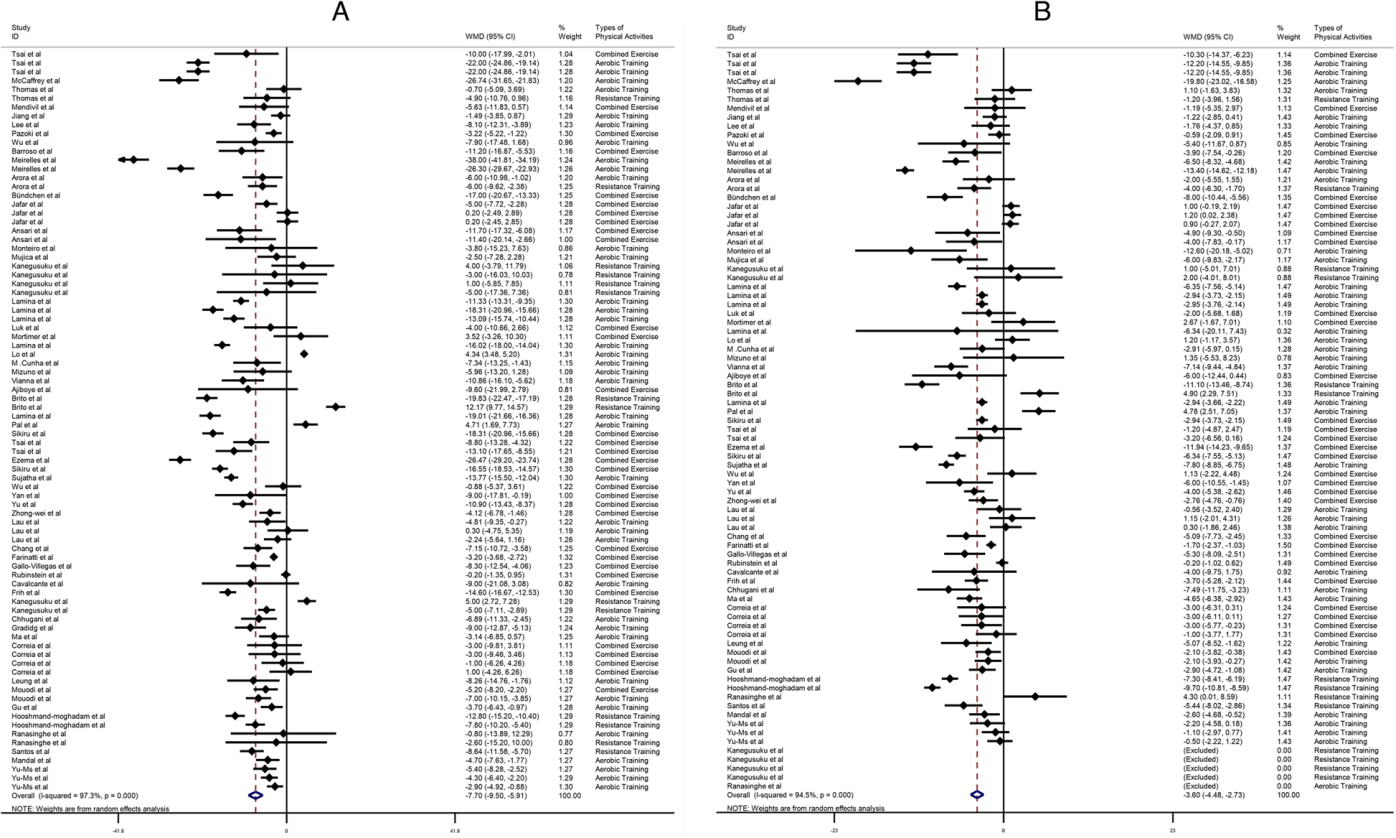
**A** Forest plot of randomized controlled trials to investigate the effect of physical activity on systolic blood pressure. **B** Forest plot of randomized controlled trials to investigate the effect of physical activity on diastolic blood pressure

A total of sixty studies, encompassing 84 effect sizes, were analyzed in this research. The studies involved a combined sample size of 11,002 participants, with 5,630 individuals classified as cases and 5,372 as controls. The primary focus of these studies was to investigate the impact of physical exercise on SBP. The results indicated that physical activity had a statistically significant impact on SBP (weighted mean difference [WMD]: -7.70 mmHg; 95% confidence interval [CI]: -9.50, -5.91; *p* < 0.001) (Fig. 2A). Nevertheless, a notable level of variability was observed (I2 = 97.3%). The subgroup analysis revealed that several factors, including type of physical activities, gender, the health status of the research participants, baseline SBP, BMI, trial duration, ethnicity, and age, accounted for the observed variability. The subgroup analysis revealed that the observed outcomes remained statistically significant when the baseline SBP was equal to or less than 120 mmHg, chose walking as a physical activity as well as in groups characterized as healthy. Healthy individuals had the greatest reduction in heterogeneity among the groups, also in people with African ethnic and male gender, they had the greatest reduction in SBP, but the heterogeneity in this group was very high.(Table 3).

**Table 3:** Subgroup analyses of physical activity on blood pressure in low and middle income countries

	NO	WMD (95%CI)	*P*-value	heterogeneity		
				P heterogeneity	I^2^	*P* between sub-groups
Subgroup analyses of physical activity on SBP						
Overall effect	84	-7.70 (-9.49, -5.91)	< 0.001	< 0.001	97.3	
Baseline SBP (mmHg)						
> 120	73	-8.15 (-10.43, -5.86)	< 0.001	< 0.001	97.5	< 0.001
≤ 120	9	-4.73 (-6.26, -3.20)	< 0.001	0.001	70.9	
Trial duration (week)						
≤ 12	50	-9.64 (-12.50, -6.79)	< 0.001	< 0.001	97.8	< 0.001
> 12	31	-4.72 (-6.37, -3.06)	< 0.001	< 0.001	91	
Age (year)						
> 50	54	-7.19 (-9.48, -4.90)	< 0.001	< 0.001	97.6	< 0.001
≤ 50	26	-8.76 (-12.34, -5.17)	< 0.001	< 0.001	96.8	
Health status						
Disease	57	-8.94 (-11.33, 6.55)	< 0.001	< 0.001	98.1	< 0.001
Healthy	26	-4.95 (-6.30, -3.60)	< 0.001	< 0.001	64.1	
Both	1	-2.24 (-5.63, 1.15)	0.196	0	0	
Gender						
Both	60	-6.84 (-8.80, -4.88)	< 0.001	< 0.001	97	< 0.001
Male	13	-14.26 (-16.24, -12.29)	< 0.001	< 0.001	84.9	
Female	11	-4.90 (-11.79, 1.98)	0.163	< 0.001	97	
Baselin BMI (kg/m^2^)						
Normal (18.5–24.9)	18	-11.75 (-14.95, -8.55)	< 0.001	< 0.001	96	< 0.001
Overweight (25–29.9)	50	-7.04 ( -9.88, -4.20)	< 0.001	< 0.001	96.5	
Obese (> 30)	5	-3.48 (-6.02, -0.95)	0.007	< 0.001	87.9	
Ethinc						
Asia	41	-6.22 (-8.52, -3.91)	< 0.001	< 0.001	96.2	< 0.001
Africa	15	-14.26 (-16.84, -11.68)	< 0.001	< 0.001	90.4	
America	27	-6.62 (-9.97, -3.28)	< 0.001	< 0.001	97.4	
Types of physical activities	84					
Resistant training	1	-3.93 (-9.32, 1.45)	0.158	< 0.001	97.1	< 0.001
Aerobic training	38	-9.17 (-12.46, -5.87)	< 0.001	< 0.001	97.9	
Combined exercise	32	-7.47 (-9.85, -5.09)	< 0.001	< 0.001	96.2	
Subgroup analyses of physical activity on DBP						
Overall effect	78	-3.60 (-4.48, -2.72)	< 0.001	< 0.001	94.5	
Baseline DBP (mmHg)						
> 80	42	-4.96 (-6.24, -3.68)	< 0.001	< 0.001	96.5	< 0.001
≤ 80	36	-1.91 (-2.62, -1.19)	< 0.001	< 0.001	63.8	
Trial duration (week)						
> 12	29	-2.36 (-3.69, -1.04)	< 0.001	< 0.001	94.1	< 0.001
≤ 12	46	-4.40 (-5.52, -3.27)	< 0.001	< 0.001	93.8	
Age (year)						
> 50	49	-3.52 (-4.56, -2.48)	< 0.001	< 0.001	94.4	< 0.001
≤ 50	26	-3.70 (-5.58, -1.82)	< 0.001	< 0.001	94.5	
Health status						
Disease	54	-4.36 (-5.46, -3.25)	< 0.001	< 0.001	95.8	< 0.001
Healthy	23	-2.05 (-2.96, -1.15)	< 0.001	< 0.001	64.3	
Both	1	0.30 (-1.85, 2.45)	0.785	0	0	
Gender						
Both	55	-3.34 (-4.52, -2.17)	< 0.001	< 0.001	94.8	< 0.001
Male	13	-5.01 (-6.43, -3.59)	< 0.001	< 0.001	93.7	
Female	10	-3.23 (-6.76, 0.29)	0.073	< 0.001	91.9	
Baselin BMI (kg/m^2^)						
Normal (18.5–24.9)	18	-4.18 (-5.37, -2.99)	< 0.001	< 0.001	90.8	< 0.001
Overweight (25–29.9)	45	-3.89 (-5.34, -2.43)	< 0.001	< 0.001	94.4	
Obese (> 30)	4	-1.95 (-3.67, -0.24)	0.025	0.001	81.3	
Ethinc						
Asia	41	-3.08 (-4.45, -1.71)	< 0.001	< 0.001	95	0.038
Africa	14	-4.50 (-5.70, -3.30)	< 0.001	< 0.001	88.4	
America	23	-4.00 (-6.07, -1.93)	< 0.001	< 0.001	95.4	
Types of physical activities	78					< 0.001
Resistant training	10	-3.03 (-6.17, -0.10)	0.058	< 0.001	95	
Aerobic training	36	-4.21 (-5.59, -2.83)	< 0.001	< 0.001	95	
Combined exercise	32	-3.00 (-4.01, -1.98)	< 0.001	< 0.001	89.9	

*Abbreviations*: *CI* Confidence interval, *WMD* Weighted mean differences, *SBP* Systolic blood pressure, *DBP* Diastolic blood pressure

### Effect of physical activity on DBP

A total of fifty-six studies, comprising 78 effect sizes, were included in the analysis. These investigations involved a combined sample size of 10,721 participants, with 5,495 cases and 5,226controls. The primary focus of these research was to investigate the impact of physical activity on DBP. The results indicate that physical activity had a substantial impact on DBP (weighted mean difference [WMD]: -3.60 mmHg; 95% confidence interval [CI]: -4.48, -2.73; *p* < 0.001) as shown in Fig. 2B. Nevertheless, a notable level of diversity was observed, as indicated by the I2 statistic of 94.5%. The subgroup analysis revealed that many factors, including type of physical activities, gender, the health status of the research participants, baseline DBP, baseline BMI, trial duration, ethnicity, and age, accounted for the observed variability. The subgroup analysis revealed that the observed outcomes continued to be statistically significant when the baseline DBP was equal to or less than 80 mmHg, chose walking as a physical activity as well as in populations without any pre-existing health conditions. People who had lower DBP equal to 80 had the greatest reduction in heterogeneity among the groups, also in people with male gender, they had the greatest reduction in DBP, but the heterogeneity in this group was very high. (Table 3).

### Sensitivity analysis and publication bias

**Fig. 3:**
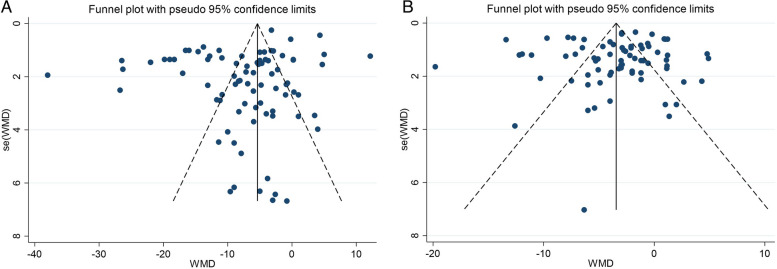
**A** Funnel plot detailing publication bias in the studies reporting the impact of physical activity on systolic blood pressure. **B** the sensitivity analysis of included studies reporting the impact of physical activity on diastolic blood pressure

The results of the sensitivity analysis revealed that the magnitude of the overall effect regarding the association between physical activity on SBP and DBP did not depend on a single study. Also, Visual inspection of the funnel plot revealed no evidence of publication bias in the studies that evaluated the effect of physical activity on SBP (Begg: 0.454) and DBP (Begg: 0.360) (Fig. 3A, B).

### Meta-regression analysis

**Fig. 4:**
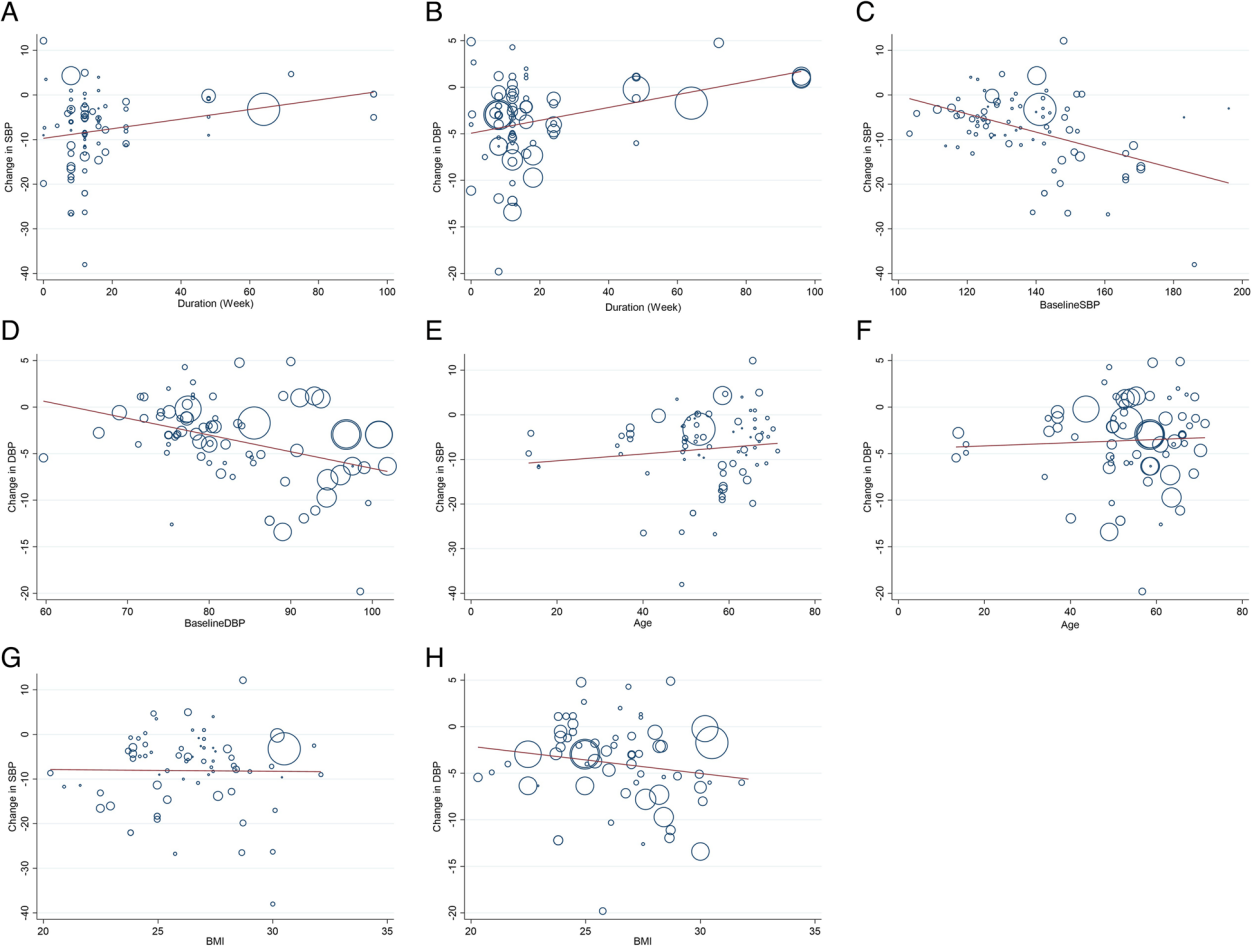
Result of meta-regression analysis for exploring the potential linear association between duration of intervention (**A**, **B**), baseline SBP and DBP (**C**, **D**), age (**E**, **F**) and BMI (**G**, **H**) with changes in SBP and DBP

Meta-regression was used to investigate the potential linear association between duration of intervention, age, BMI, baseline SBP, and DBP with changes in SBP and DBP. Accordingly, meta-regression analysis did reveal a significant association between the duration of intervention and changes in SBP (P _linearity_ = < 0.001) and DBP (P _linearity_ = 0.004). The findings indicate that the longer the intervention period, the less impact it has on both SBP and DBP levels. In other words, the effectiveness of the intervention decreases as its duration increases.(Fig. 4A, B). Also, there was a significant association between baseline SBP and changes in SBP (P linearity = < 0.001) and between baseline DBP and changes in DBP (P_linearity_ = 0.001) (Figs. 4C, D). Based on the graphs, it can be observed that as the baseline BP level increases, there is a corresponding increase in the blood pressure level during physical activity. In other words, the higher the baseline BP, the greater decrease in the level of blood pressure was observed. However, meta-regression analysis did not show any significant association between age and changes in SBP (P_linearity_ = 0.282), DBP (P_linearity_ = 0.631), and BMI with changes in SBP (P_linearity_ = 0.871) and DBP (P_linearity_ = 0.208) (Figs. 4E–H).

### Grading of evidence

The GRADE protocol was used to assess the certainty of the evidence (Table 4). Accordingly, studies investigating the effect of physical activity on SBP and DBP were regarded as moderate quality due to the high heterogeneity between studies.

**Table 4:** GRADE profile of physical activity for SBP and DBP

Quality assessment						Summary of findings		Quality of evidence
Outcomes	Risk of bias	Inconsistency	Indirectness	Imprecision	Publication Bias	Number of intervention/control	WMD (95%CI)	
SBP	No serious limitation	Very serious limitation^a^	No serious limitation	No serious limitation	No serious limitation	5630/5372	-7.70 (-9.50, -5.91)	⊕ ⊕ ⊕ ◯ Moderate
DBP	No serious limitation	Very serious limitation^b^	No serious limitation	No serious limitation	No serious limitation	5495/5226	-3.60 (-4.48, -2.73)	⊕ ⊕ ⊕ ◯ Moderate

^a^The test for heterogeneity is significant, and the I^2^ is high, 97.3%

^b^The test for heterogeneity is significant, and the I^2^ is high, 94.5%

## Discussion

We conducted a meta-analysis that indicates a relation between blood pressure (SBP and DBP) and physical activity (PA). Our findings also, suggest that PA is a good lifestyle intervention for reducing BP. This study represents the first meta-analysis conducted to examine the effects of PA on blood pressure across low and middle-income countries (LMICs), as far as our current understanding allows us to ascertain. The rigorous evaluation of the outcomes of this investigation is crucial owing to the significant heterogeneity seen throughout the analyses. To account for this heterogeneity, subgroup analyses were performed based on type of physical activities (Resistant training, Aerobic training,Combined exercise), trial duration (≤ 12 vs. > 12 weeks), age (≤ 50 vs. > 50 years), health status (healthy vs. disease vs. both), gender (male, female, both), ethnicity (Asian vs. African vs. American), baseline BMI (normal (18.5–24.9) vs. overweight (25–29.9) vs. obese (≥ 30)), baseline SBP (≤ 120 vs. > 120 mmHg), and baseline DBP (≤ 80 vs. > 80 mmHg). Subgroup analysis showed that the results remained significant when baseline SBP ≤ 120 mmHg and in healthy populations for SBP and the results remained significant when baseline DBP ≤ 80 mmHg and in healthy populations in DBP, respectively. The findings of our study align with other research indicating that engaging in leisure-time PA has the potential to decrease both SBP and DBP levels [96]. Nevertheless, it is important to acknowledge a constraint of this study, namely the limited number of studies that were encompassed, potentially impeding the extent to which the findings can be applied to a broader population [96]. Moreover, another recent study supports the perspective that a higher intensity of PA is associated with larger reductions in DBP [97]. The primary focus of our analysis was on LMICs, and it encompassed a substantial number of RCTs. In contrast, previous investigations have examined this correlation in smaller cohorts and did not specifically target this particular demographic. Different kinds of medication are used in the treatment of hypertension and they can lower the BP in different ways. The ACE enzyme facilitates the transformation of angiotensin I to angiotensin II, which results in heightened blood pressure. ACEIs impede this process, whereas ARBs obstruct the receptors. CCBs curtail vascular resistance by hindering calcium channels. Diuretics decrease blood pressure by impeding sodium reabsorption at different segments of the nephron. Thiazides target the distal convoluted tubule to check the sodium-chloride cotransporter [98]. However, the role of PA in reducing BP can be as important as pharmacological treatment [16]. The precise mechanisms via which PA mitigates the development of hypertension are still uncertain and subject to debate. This is mostly attributed to the complex and multifaceted character of hypertension, as well as the ambiguous ways in which several contributing factors interact with each other. According to the research conducted by Millar and colleagues (Millar PJ et al.), it is proposed that the decrease in resting blood pressure (BP) resulting from isometric exercise training necessitates modifications in either one or both of the factors that determine mean arterial pressure, namely cardiac output and total peripheral resistance [99]. Following mechanisms can be mentioned: PA induces a vasodilatory response that widens blood vessels and diminishes their constriction [100, 101]. This response includes decreasing cardiac output, sympathetic nervous system activity, levels of plasma norepinephrine, and total peripheral resistance [102, 103]. One of the other mechanisms is improving endothelial function which can be compromised in individuals with hypertension [102, 104–106]. Based on a comprehensive study of existing research studies and a synthesis of their findings, it has been determined that aerobic endurance training has the capacity to lower BP by diminishing vascular resistance. This effect is believed to be mediated via the involvement of the sympathetic nervous system and the renin-angiotensin system [107]. Furthermore, hyperinsulinemia and insulin resistance can lead to hypertension. This happens because insulin causes the body to hold onto sodium, makes the nervous system more active, and causes the proliferation of muscles inside blood vessels [102, 108]. Exercise improves insulin sensitivity, providing another possible mechanism for its antihypertensive effect [102, 109]. Additionally, PA may decrease plasma viscosity, leading to less resistance in blood vessels and improving blood flow [102]. Apart from what was previously mentioned, doing exercise can lower the chance of getting high BP by helping maintain a healthy weight and reducing fat in the body which is the main reason for high BP [102, 110–113]. Besides affecting the constriction and relaxation of blood vessels, exercise also helps to reduce BP by decreasing the levels of oxidative stress and inflammation [100]. According to a recent systematic review and meta-analysis of randomized clinical trials, Strength training can help reduce BP by improving cardiovascular function, increasing muscle strength and endurance, promoting weight loss, and reducing peripheral vascular resistance [114]. It can also improve insulin sensitivity and glucose metabolism, which can contribute to a reduction in BP [114]. One possible explanation for this finding could be that strength training makes the body produce more nitric oxide, which in turn leads to the vasodilation of blood vessels [114].

### Strengths and limitations

There are some noteworthy strengths inherent in this study that warrant acknowledgment. This study encompasses a thorough investigation and examination of the existing research about the influence of physical activity on blood pressure. The search technique employed in this study was comprehensive and encompassed a variety of databases, hence reducing the likelihood of inadvertently omitting relevant studies. Furthermore, we have incorporated data from 19 LMICs, so enhancing the generalizability of our findings to all nations of similar economic status. Furthermore, our investigations did not identify any potential sources for conducting sensitivity analysis. Nevertheless, it is important to acknowledge the presence of several limitations in our study that may have been mitigated. The RCTs that were included in our investigation exhibited variability in terms of both sample size and length of the intervention. Moreover, it is worth noting that the individuals in each study exhibited varying health statuses, as well as diverse dietary and lifestyle patterns, which may have influenced the outcomes observed.

## Conclusion

Recent research consistently validates the connection between PA and BP. Given the rise in high BP cases in LMICs, notable emphasis has been placed on the fact that our comprehensive analysis also verifies the substantial impact of PA in lowering BP within these countries.

### Supplementary Information

Supplementary Material 1.

## Data Availability

The datasets used and/or analyzed during the current study are available from the corresponding author upon reasonable request.
